# Babesiosis in Lower Hudson Valley, New York, USA

**DOI:** 10.3201/eid1705.101334

**Published:** 2011-05

**Authors:** Julie T. Joseph, Sumith S. Roy, Navid Shams, Paul Visintainer, Robert B. Nadelman, Srilatha Hosur, John Nelson, Gary P. Wormser

**Affiliations:** Author affiliations: New York Medical College, Valhalla, New York, USA (J.T. Joseph, S.S. Roy, N. Shams, R.B. Nadelman, S. Hosur, J. Nelson, G.P. Wormser);; Baystate Health System, Springfield, Massachusetts, USA (P. Visintainer)

**Keywords:** babesiosis, parasites, Lower Hudson Valley, New York, tick-borne disease, transfusion, research

## Abstract

Although Lyme disease has been endemic to parts of the Lower Hudson Valley of New York, United States, for >2 decades, babesiosis has emerged there only since 2001. The number of Lower Hudson Valley residents in whom babesiosis was diagnosed increased 20-fold, from 6 to 119 cases per year during 2001–2008, compared with an ≈1.6-fold increase for the rest of New York. During 2002–2009, a total of 19 patients with babesiosis were hospitalized on 22 occasions at the regional tertiary care center. Concurrent conditions included advanced age, malignancies, splenectomy, and AIDS. Two patients acquired the infection from blood transfusions and 1 from perinatal exposure, rather than from a tick bite. One patient died. Clinicians should consider babesiosis in persons with fever and hemolytic anemia who have had tick exposure or have received blood products.

## Abstract 2

Cases were associated with tick bites and receipt of blood products.

Babesiosis is a tick-borne infection of erythrocytes. *Babesia microti*, the most common cause of babesiosis in North America, is transmitted by *Ixodes scapularis* ticks, which also transmits *Borrelia burgdorferi*, the cause of Lyme disease, and *Anaplasma phagocytophilum*, the cause of human granulocytic anaplasmosis (HGA) (1,2). Babesiosis, however, does not occur in all Lyme disease–endemic areas (1). Although Lyme disease has been highly endemic to parts of the Lower Hudson Valley (LHV) of New York in the United States since the mid-1980s, the first indigenous case of babesiosis did not occur there until 2001 (3).

To better characterize the recent emergence of babesiosis in this region, we reviewed data for 2001–2008 on I. scapularis tick–transmitted infections in the 7 counties that make up the LHV. These counties are located immediately north of New York City. In addition, we reviewed the medical records of patients with babesiosis who were hospitalized during January 1, 2002–December 31, 2009, at the Westchester Medical Center (WMC), the sole tertiary care medical center in the LHV.

## Methods

### Reported Babesiosis Cases in the LHV

For this report, we defined the LHV as Westchester, Putnam, Dutchess, Orange, Rockland, Ulster, and Sullivan counties (4). Cases of babesiosis, Lyme disease, and HGA in this region were tabulated on the basis of statistics on reportable diseases available on the New York State Department of Health (NYSDOH) website (5). Cases listed as ehrlichiosis were assumed to be a surrogate for HGA in this region. For purposes of surveillance by the NYSDOH during the period reviewed, a diagnosis of babesiosis was considered confirmed when 1) a clinically compatible illness occurred in conjunction with identification of *Babesia* spp. parasites on blood smear or a positive immunoglobulin G (or total antibody) *Babesia* spp. serologic titer of >256 (with testing confirmed by NYSDOH), or 2) in the absence of a clinically compatible illness, *Babesia* spp. parasites were present on blood smear (5).

### Patients Hospitalized with Babesiosis at WMC

WMC is located in Valhalla, Westchester County, New York. We retrospectively reviewed medical records of patients with babesiosis documented by peripheral blood smear who were hospitalized at WMC during January 1, 2002–December 31, 2009. Case ascertainment was based on review of microbiology and infectious diseases records. For the 2 patients who had >1 hospitalization for babesiosis, we included data for only the first hospitalization. Complete records were available for all but 1 patient; partial records were available for that patient. The Institutional Review Board at New York Medical College approved the medical records review.

### Statistical Methods

Continuous variables were described with means and standard deviations. Categorical variables were described with frequencies and percentages, and differences were compared with the Fisher exact test (2-tailed). Relative risk estimates over time and among counties were computed by using Poisson regression adjusting for population size. A p value <0.05 was considered significant.

## Results

**Figure 1:**
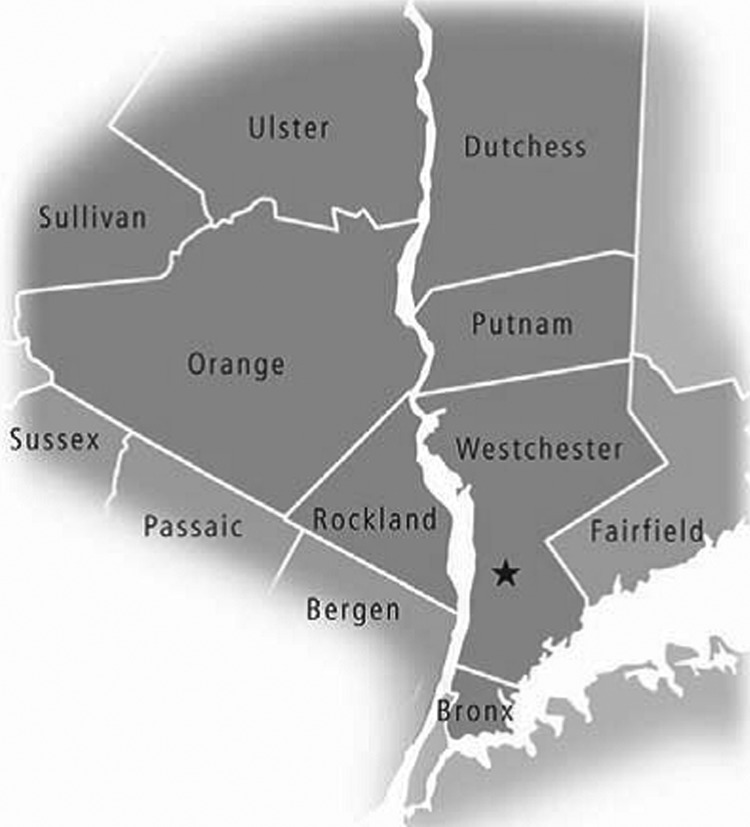
Map of the Lower Hudson Valley of New York, USA. Westchester, Putnam, and Dutchess Counties are east of the Hudson River, and Orange, Rockland, Ulster and Sullivan Counties are west of the Hudson River. The star indicates the site of the Westchester Medical Center. Permission for use of this image granted from the Westchester Institute for Human Development on July 23, 2010.

The LHV comprises 4 counties west of the Hudson River (Rockland, Orange, Sullivan, and Ulster) and 3 counties east of the Hudson River (Westchester, Putnam, and Dutchess) (Figure 1). Westchester County is located immediately north of the Bronx, New York.

**Figure 2:**
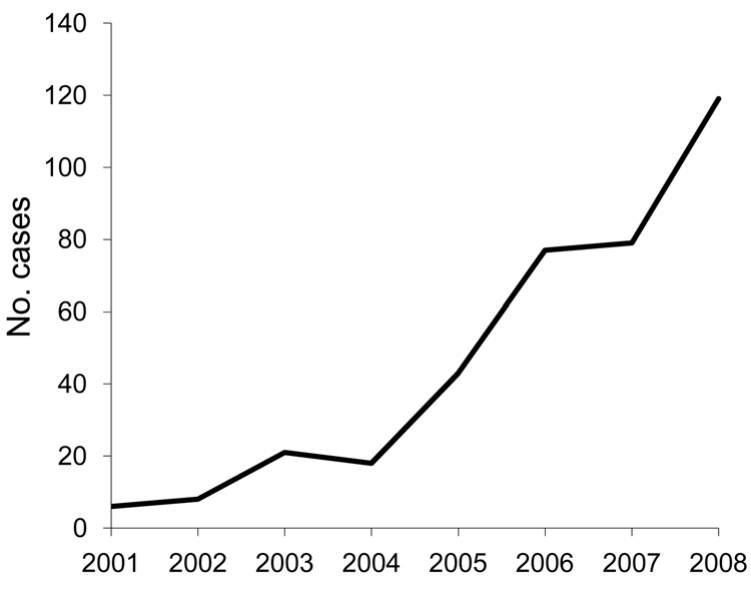
Annual number of reported babesiosis cases, Lower Hudson Valley, New York, USA, 2001–2008.

Babesiosis has been a reportable disease in New York since 1986. According to statistics compiled by NYSDOH (5), the number of cases of babesiosis diagnosed in residents of the 7-county LHV increased nearly 20-fold from 6 per year to 119 per year during 2001–2008 (Figure 2), with an average increase in incidence of 48.7% per year (95% confidence interval [CI] 40.6%–57.2%) (Table 1) (5,6). In the rest of the state, the number of cases increased only ≈1.6-fold during the same period (from 89 cases in 2001 to 142 cases in 2008) (5).

**Table 1:** Babesiosis cases reported to the New York State Department of Health, Lower Hudson Valley, New York, USA, 2001–2008

Area (2008 population)	2001	2002	2003	2004	2005	2006	2007	2008
West of Hudson River (936,051)	0	0	1	2	5	7	5	15
Rockland County (298,545)	0	0	0	1	0	2	0	3
Orange County (379,647)	0	0	1	1	1	5	5	7
Sullivan County (76,189)	0	0	0	0	1	0	0	1
Ulster County (181,670)	0	0	0	0	3	0	0	4
East of Hudson River (1,346,065)	6	8	20	16	38	70	74	104
Dutchess County (292,878)	2	4	6	7	23	42	44	62
Putnam County (99,244)	1	0	1	0	2	3	1	6
Westchester County (953,943)	3	4	13	9	13	25	29	36

Although the number of babesiosis cases increased on both sides of the river, 104 (87.4%) of 119 reported cases in 2008 occurred in residents of counties east of the Hudson River (Table 1). The 104 cases in the 3 counties east of the Hudson River, with a total population of 1,346,065 (6), corresponds to 7.7 cases per 100,000 residents, compared with 15 cases among a total population of 936,051 or 1.6 cases per 100,000 for the 4 counties west of the Hudson River (relative risk [RR] 4.82, 95% CI 2.79–8.92; p<0.001). In the 3 counties east of the river, Dutchess County accounted for 62 of the babesiosis cases in 2008 (21.2/100,000), Westchester County for 36 cases (3.8/100,000), and Putnam County for 6 cases (6.0/100,000); thus, the prevalence of babesiosis in 2008 was significantly greater for Dutchess County than for Westchester County (RR 5.61, 95% CI 3.72– 8.46; p<0.001) or for Dutchess than for Putnam County (RR 3.53, 95% CI 1.51–8.09; p = 0.003). No significant difference was detected between Putnam and Westchester Counties (RR 1.60, 95% CI 0.68–3.81; p = 0.28) (5,6).

For purposes of comparison, in 2001, a total of 2,584 Lyme disease cases were reported from the LHV, compared with 4,609 in 2008, representing a <2-fold increase; 78 ehrlichiosis (HGA) cases were reported in 2001, compared with 213 in 2008, a <3-fold increase (5). In 2008, 2,369 (51.4%) of the 4,609 reported Lyme disease cases occurred in residents of counties east of the Hudson River, compared with 186 (87.3%) of 213 reported ehrlichiosis (HGA) cases.

### Hospitalized Patients with Babesiosis

Coincident with the emergence of babesiosis in the LHV, the number of patients hospitalized at WMC with this infection also markedly increased. Nineteen patients (18 adults) were hospitalized with babesiosis at WMC on 22 occasions from 2002 through 2009. All 19 patients were residents of LHV; 15 (79%) resided in Westchester County, 2 in Dutchess County, and 1 each in Orange and Putnam Counties.

The only child affected was a 6-week-old infant who acquired *B.* *microti* infection perinatally; a detailed case history for this patient will be reported elsewhere. For 2 of the 18 cases in adults, transfusion of infected blood products was believed to have been the route of infection; 1 of these cases is described in more detail elsewhere (7). Fifteen (94%) of the 16 other adult patients had potential tick exposure in the LHV (tick exposure is defined as exposure to outdoor environments where ticks are likely to reside); for 10 (67%) of these patients, this was the only known tick exposure within 30 days before onset of symptoms. Of the 16, however, only 3 (19%) actually recalled a tick bite within this 30-day period.

All 18 adult patients had a positive peripheral blood smear for *Babesia* spp. parasites (Table 2). Of the 8 patients who were tested for *B. microti* DNA by PCR, all had positive results. All but 2 of the patients were admitted during May–October. One patient was admitted in December, and the other was admitted in January. The patient who sought care in December had a tick bite 1 month before admission. Thirteen (72%) patients were men; the mean age was 54.1 years (range 21–95 years). Mean time from onset of symptoms to diagnosis was 13.6 days (median 11 days, range 3–33 days).

**Table 2:** Selected demographic and clinical features and laboratory test results for 18 adults with babesiosis hospitalized at Westchester Medical Center, Valhalla, New York, USA, 2002–2009*

Characteristic	Value
Mean age, y, ± SD (range)	54.1 ± 20.1 (21–95)
Male, no. (%)	13 (72.2)
Mean time from symptom onset to diagnosis, d, ± SD (range)	13.6 ± 9.28 (3–33)
Recollection of tick bite within 30 d, no. (%), n = 16	3 (18.8)
Temperature >38°C, no. (%)	15 (83.3)
Splenomegaly, no. (%), n = 13	2 (15.4)
Mean initial parasitemia, %, ± SD (range), n = 17†	4.49 ± 4.57 (0.01–14)
Mean highest level of parasitemia, %, ± SD (range), n = 17†	5.34 ± 5.79 (0.05–18)
Mean initial leukocyte count × 109/L,± SD (range), n = 17	7.2 ± 3.38 (3.2–15.4)
Lymphocyte count <1,000 ×106/L, no. (%), n = 12	5 (41.6)
Mean hemoglobin minimum, g/dL,± SD (range)	8.2 ± 1.98 (3.5–11.1)
Mean platelets minimum, × 109/L, ± SD (range)	110.8 ± 139.2 (19–615)
Platelets minimum <150 × 109/L, no. (%)	16 (88.9)
Mean initial erythrocyte sedimentation rate, mm/h,± SD (range), n = 9	76.7 ± 33.3 (32–138)
Mean initial lactate dehydrogenase, U/L, ± SD (range), n = 15	931.5 ± 562 (229–2074)
Mean initial aspartate aminotransferase, U/L, ± SD (range)	237.7 ± 366.9 (19–1450)
Initial aspartate aminotransferase >30 U/L, no. (%)	14 (77.8)
Mean initial alanine aminotransferase, U/L, ± SD (range)	110.2 ± 111 (16–433)
Initial alanine aminotransferase >40 U/L, no. (%)	13 (72.2)
Mean initial total bilirubin, mg/dL, ± SD (range)	3.4 ± 5.59 (0.4–24.6)
Initial total bilirubin >1.2 mg/dL, no. (%)	10 (55.6)
Mean serum sodium minimum, meq/L, ± SD (range), n = 17	127.6 ± 10.1 (94–139)
Mean creatinine maximum , ng/mL, ± SD (range), n = 17	1.3 ± 0.59 (0.7–2.7)

*Data were obtained from all 18 patients unless otherwise indicated.  †For 1 patient with a positive smear, the level of parasitemia is unknown.

Five (28%) patients had had a splenectomy before the babesiosis diagnosis, 2 (11%) had AIDS, and 5 (28%) had malignancies (2 of whom were among the 5 patients who had splenectomies). Of the 5 patients with malignancies, 1 had acute myelogenous leukemia and had received a stem cell bone marrow transplant, 2 patients had B-cell follicular lymphoma (and had been treated with rituximab), 1 had a teratoma, and 1 had renal cell carcinoma. Of the 8 patients <50 years of age, 5 (63%) were potentially immunocompromised because of malignancy, splenectomy, or AIDS.

Common symptoms or signs were fever (temperature >38°C) (83%), headache (39%), malaise (33%), and chills (28%); splenomegaly was present in 2 (15%) of the 13 patients with an intact spleen. Frequent laboratory findings included anemia, thrombocytopenia, and abnormal liver function tests (Table 2). All 15 patients for whom a lactate dehydrogenase level was available had a value above the upper reference limit (221 U/L). Reticulocytes varied from 1.1% to 19.9% in 12 patients (median 3.1%; reference 0.5%–1.5%). Haptoglobin level was <20 mg/dL in all 10 patients who were tested (reference 26–85 mg/dL).

Eleven patients were treated with azithromycin and atovaquone; a rash to azithromycin developed in 1 patient, and the drug regimen was changed to clindamycin and atovaquone. In another patient, a rash to atovaquone developed, and clindamycin and quinine was prescribed. Six patients were initially treated with clindamycin and quinine; adverse reactions to quinine developed in 3. In 1 patient, QT prolongation developed, and in 2 patients, hearing loss developed. One patient was initially treated with clindamycin and atovaquone. Eight (44%) patients required blood transfusion for anemia, and 3 (17%) received erythrocyte exchange as adjunctive therapy.

Length of hospital stay ranged from 3 to 73 days (median 8 days). One patient had left upper quadrant pain and splenic rupture and was treated conservatively without surgery. The 1 death occurred in a 95-year-old patient in whom shock and respiratory failure developed and who required admission to the intensive care unit. Another patient required ventilator support. In 15 (83%) patients, infection resolved with a single course of antimicrobial drugs. Illness recurred in 2 patients but resolved after a subsequent and more prolonged course of antimicrobial drug treatment (the 2 latter patients have been included in previous reports [7–9]).

## Discussion

As of 2008, babesiosis cases in residents of the LHV accounted for 45.6% of the 261 cases reported in New York (5). Testing of selected *I. scapularis* ticks by PCR in 2002 showed positive results for *B. microti* in tick pools collected in Dutchess and Westchester Counties (5). A more recent study of 154 adult *I. scapularis* ticks collected in 2008 from 2 locations in Westchester County identified 24 (15.6%) ticks that were infected with *B. microti* according to PCR, compared with 34 (25.8%) of 132 adult ticks collected from 3 locations in Suffolk County, in Long Island, New York (p<0.04) (10); babesiosis has been indigenous to Suffolk County since 1975, with 95 cases reported there in 2008 alone (5). These infection rates, however, should be interpreted cautiously because an unknown proportion of positive findings may have resulted from detection of *B. odocoilei* in the ticks evaluated, rather than *B. microti*. *B. odocoilei*, which is not regarded as a human pathogen, infects deer ticks more frequently than does *B. microti* in sites where these piroplasms coexist (11).

There are 2 prior reports of hospitalized patients in New York with babesiosis. One report published in 1998 described 139 adults with babesiosis hospitalized during 1982–1993 (12). More than 90% of these patients resided in Suffolk County; only 2 resided in Westchester County. The other report, published in 2001, described 34 adults and children with babesiosis hospitalized at 2 tertiary care centers in Suffolk County (13). The latter patients were hospitalized over 13 consecutive years, but the exact years were not specified. The general clinical and laboratory features of babesiosis in these 2 case series were similar to those observed in the patients in our study. Most patients had a nonspecific febrile illness associated with hemolytic anemia, thrombocytopenia, and abnormal liver function test results. Of the 139 patients in the 1998 series, 16 (11.7%) had had a splenectomy (12), as did 8 (27%) of the 30 adults in the 2001 report (13), but in neither of the 2 earlier reports were any patients identified as having lymphoma and receiving treatment with rituximab (9), receiving a transplantation, or having AIDS. Thus, our case series presumably included more patients now recognized to be at high risk for relapse of infection (9). The 5.6% case-fatality rate in our study, however, is slightly lower than the 6.5% in the 1998 report (12) and the 8.8% in the 2001 report (13). Unlike the 2 prior case series, 2 (11%) of the patients in our study were believed to have been infected through receipt of an infected blood product (7), which provides further evidence of the growing importance of this route of transmission (14–18).

Six (33%) of the patients reported here had serologic evidence of Lyme disease, but this finding may overestimate the frequency of coinfection because only 1 had an objective clinical manifestation of Lyme disease (erythema migrans). Among the adult ticks collected in Westchester County in 2008 (10), 79.2% of those infected with *Babesia* spp. were also infected with *B. burgdorferi*, which reinforces the need to consider the possibility of concomitant Lyme disease in patients from the LHV in whom babesiosis is diagnosed.

How *B. microti* found its way from areas to which this microorganism is endemic into the I. scapularis tick population of the LHV is unclear. Evidence suggests that babesiosis is also emerging as a human pathogen in contiguous geographic areas of western Connecticut (19,20). The principal animal reservoir for *B. microti* is the white-footed mouse, *Peromyscus leucopus* (1). Other reservoirs include voles and shrews. These animals are not likely to travel great distances, which suggests that movement of these animals is an unlikely explanation for the emergence of babesiosis in the LHV.

Babesiosis is an emerging infectious disease in the LHV of New York with the potential to cause serious illness and death, especially in highly immunocompromised patients. Clinicians should consider this diagnosis in persons with fever and hemolytic anemia who have been exposed to ticks or have received blood products.
